# Association between the unemployment rate and inpatient cost per discharge by payer in the United States, 2005–2010

**DOI:** 10.1186/1472-6963-14-378

**Published:** 2014-10-13

**Authors:** Jared Lane K Maeda, Rachel Mosher Henke, William D Marder, Zeynal Karaca, Bernard S Friedman, Herbert S Wong

**Affiliations:** Mid-Atlantic Permanente Research Institute, Kaiser Permanente (formerly), 2101 East Jefferson Street, Rockville, MD 20852 USA; Truven Health Analytics, 150 Cambridge Park Drive, Cambridge, MA 02140 USA; Center for Delivery, Organization, and Markets, Agency for Healthcare Research and Quality, 540 Gaither Road, Rockville, MD 20850 USA

## Abstract

**Background:**

Several reports have linked the 2007–2009 Great Recession in the United States with a slowdown in health care spending and decreased utilization. However, little is known regarding how the recent economic downturn affected hospital costs per inpatient stay for different segments of the population. The purpose of this study was to examine the association between changes in the unemployment rate and inpatient cost per discharge for Medicare and commercial discharges.

**Methods:**

We used retrospective data at the Core Based Statistical Area (CBSA)-level from 46 states that contributed to the Healthcare Cost and Utilization Project State Inpatient Databases from 2005 to 2010. Unemployment data was derived from the American Community Survey. An instrumental variable two-stage least squares approach with fixed- or random-effects was used to examine the association between unemployment rate and inpatient cost per discharge by payer because of potential endogeneity.

**Results:**

The marginal effect of unemployment was associated with an increase in inpatient cost per discharge for both payers. A one percentage point increase in the unemployment rate was associated with a $37 increase for commercial discharges and a $49 increase for Medicare discharges.

**Conclusions:**

We find evidence that the inpatient cost per discharge is countercyclical across different segments of the population. The underlying mechanisms by which unemployment affects hospital resource use however, might differ between payer groups.

## Background

The economic shock from the 2007–2009 Great Recession in the United States, in part, contributed to the slowest annual rate of increase in health care spending in nearly fifty years. In 2010, overall health care spending grew by only 3.9 percent and spending on hospital inpatient services slowed to 4.9 percent [1].

Recessions also appear to impact health care utilization. Previous studies indicate that health care utilization decreases during periods of economic contraction because of declining demand for medical care [2]. More specifically, economic downturns appear to affect hospital utilization. For example, a survey of short-term acute care hospitals found that 72% of respondents reported a decline in the volume of elective procedures as a result of the recession [1]. Additionally, although there was a modest growth in inpatient admissions in 2008 [3], there was a moderate to significant decrease in inpatient admissions in 2009 and 2010 [1, 4]. Economic downturns might also influence a provider’s decision to admit specific subgroups of patients to the hospital [5].

While nonelderly adults who lose insurance coverage because of involuntary job loss are most likely to reduce their utilization, individuals who are continuously insured during economic downturns may reduce discretionary spending on health care services and take fewer preventive measures in response to the fear of job loss, declining household income, and greater economic insecurity [6]. Although the health care patterns of the working age, commercially insured population are sensitive towards economic conditions, the over age 65 year old Medicare population should be relatively insulated from fluctuations in the business cycle. Thus, the impact of unemployment on health care utilization might differ between payer groups.

Although previous studies have reported a decreasing trend in hospital utilization during the 2007–2009 Great Recession, little is known regarding how the recent economic downturn affected hospital costs per inpatient stay for different segments of the population. Commercially insured individuals may have postponed their medical care and became sicker resulting in more costly admissions. In contrast, the recession may have had little to no impact on the inpatient cost per discharge for the Medicare population.

In this study, we sought to examine the association between annual changes in Core Based Statistical Area (CBSA)-level unemployment rate and inpatient cost per discharge for all-payers and payer-specific discharges (Medicare and commercially insured). We focused on the CBSA-level because there may be greater variation in the unemployment rate and cost per discharge within states than between states. This study might provide a better understanding of the impact of the recent economic slowdown on inpatient costs per stay and whether the effects were the same for both the working age and Medicare-eligible populations.

## Methods

### Data sources

We used all-payer data from 46 states that contributed to the Healthcare Cost and Utilization Project (HCUP) State Inpatient Databases (SID) from 2005 to 2010. The HCUP SID include the largest collection of longitudinal hospital care data in the United States and contain information from the universe of inpatient discharge records in participating states [7]. HCUP databases are publicly available for all researchers and can be purchased through the HCUP Central Distributor. We selected our study period to include the interval just prior to and following the 2007–2009 Great Recession. We examined the effect of the annual changes in the CBSA-level unemployment rate on the inpatient cost per discharge over time for all-payers and for Medicare and commercial discharges.

### Geographic areas

CBSAs were selected as the geographic unit of analysis because they represent the universe of metropolitan and micropolitan areas in the United States, and they have been used in previous studies to examine geographic variations [8–11]. CBSAs include a core area with a substantial population that, together with their adjacent communities, contains a high degree of economic and social integration [8]. Metropolitan statistical areas are defined as having at least one urbanized area of 50,000 or more residents. Micropolitan statistical areas are defined as having fewer than 50,000 residents, but include at least one urban cluster of at least 10,000 [6]. Although previous studies on geographic variation have used the Hospital Referral Region (HRR) which is derived using the Medicare population, HRR-level analyses may not be appropriate to study the non-Medicare population because health care utilization patterns may differ between Medicare and the commercially insured.

The HCUP inpatient data from discharges at community, acute care hospitals were aggregated to 459 CBSAs based on patient ZIP code. The data included both metropolitan and micropolitan statistical areas. In instances where the CBSA boundary overlapped between states that did and did not contribute SID data, we included those CBSAs for which 99% or more of the CBSA population resided in the state that contributed the HCUP data.

### Unemployment rate

The unemployment rate was derived from the American Community Survey (ACS). The unemployment rate is the annual the number of people in the civilian workforce age 16 and over who are unemployed divided by the number of civilians in the workforce multiplied by 100 [12].

### Inpatient cost per discharge

Hospital-level total inpatient costs were derived from inpatient charges using the HCUP cost-to-charge ratios [13]. The costs were adjusted for cost of living by multiplying the hospital-level total inpatient costs by the area wage index. Costs were also adjusted to 2010 dollars using the gross domestic product (GDP) deflator. The inpatient cost per discharge was then calculated by taking the sum of the total hospital inpatient costs in a CBSA divided by the total number of discharges for each CBSA.

All-payer inpatient cost per discharge was calculated using patients 40 years and older. Payer-specific cost per discharge was calculated by using patients aged 40–64 years for commercial insurance and patients aged 65 years and older for the Medicare population.

### Payer

We used the primary expected payer on the discharge record to identify the payer source. *Medicare* was assigned if the primary expected payer was Medicare fee-for-service (FFS) or Medicare Managed Care and age was 65 years or older. *Commercial* was assigned to discharge records that had a primary expected payer of indemnity, health maintenance organization (HMO), preferred provider organization (PPO), or point of service (POS) insurance plans and age was 40–64 years. *All-payers* included discharges from Medicare and commercial as well as any other payers, including self-pay and other government programs that was 40 years and older.

### Model covariates

We adjusted for patient, population, market, and hospital quality of care characteristics that may also influence the cost per discharge. The patient characteristics of age, sex, and comorbidities were derived from the SID. The comorbidities were based on the Elixhauser comorbidity index [14]. Population characteristics were obtained from the American Community Survey and the Area Health Resource File (AHRF) and included sociodemographic characteristics. Market characteristics such as the number of primary care physicians per capita and number of emergency department (ED) visits per capita were derived from the American Hospital Association (AHA) Annual Survey and AHRF. Based on previous work, we created a measure of high technology hospitals defined as those hospitals that reported having at least 6 of 8 high tech services in the AHA Annual Survey [15–17]. We also included hospital quality of care in our models by using a composite score of the Centers for Medicare and Medicaid Services (CMS) Hospital Compare performance measures.

The Herfindahl-Hirschman Index (HHI) was used to define the intensity of market competition and it was derived from the Hospital Market Structure (HMS) file developed for HCUP. The HHI is scaled from 0–100, where zero represents many competitors in a market and 100 represents a monopoly.

We included the proportion of discharges from a CBSA different from the hospital’s CBSA to measure the patterns of patient flow and market competitiveness. The entire list of covariates used in the regression models is provided in Tables 1 and 2.

**Table 1:** Average CBSA patient characteristics by Payer, 2005 and 2010*

	All payers		Medicare		Private insurance ^†^	
	2005	2010	2005	2010	2005	2010
	n = 393	n = 455	n = 393	n = 455	n = 393	n = 455
	Mean	Mean	Mean	Mean	Mean	Mean
**Cost per discharge**	11,097	12,712	11,110	12,570	11,099	13,173
***Patient characteristics***						
**Age**	66.8	66.9	77.9	77.9	53.0	53.8
**Sex**						
Female (%)	0.55	0.54	0.58	0.57	0.53	0.52
**% of Discharges with comorbid conditions**						
Addictive disorders (combined)	0.15	0.21	0.11	0.15	0.14	0.20
Diabetes (combined)	0.24	0.28	0.25	0.30	0.18	0.21
General blood disorders (combined)	0.21	0.28	0.26	0.34	0.13	0.18
General cancers (combined)	0.03	0.03	0.03	0.04	0.03	0.04
Rheumatoid arthritis/collagen vascular diseases	0.03	0.03	0.03	0.04	0.02	0.02
Congestive heart failure	0.10	0.10	0.15	0.15	0.03	0.03
Chronic obstructive pulmonary disease	0.21	0.22	0.24	0.24	0.13	0.14
Hypertension (combined uncomplicated and complicated)	0.51	0.60	0.58	0.67	0.41	0.48
Hypothyroidism	0.10	0.14	0.13	0.17	0.07	0.09
Liver disease	0.02	0.03	0.01	0.01	0.02	0.03
Fluid and electrolyte disorders	0.19	0.25	0.22	0.28	0.12	0.17
Other neurological disorders	0.06	0.08	0.08	0.10	0.02	0.04
Obesity	0.07	0.11	0.04	0.08	0.09	0.15
Paralysis	0.02	0.03	0.02	0.03	0.01	0.01
Renal failure	0.06	0.14	0.08	0.18	0.02	0.05
Solid tumor without metastasis	0.02	0.02	0.03	0.03	0.02	0.02
Valvular disease	0.04	0.04	0.06	0.06	0.02	0.02
Weight loss	0.03	0.05	0.03	0.05	0.03	0.05

^†^Commercial includes non-maternal adults aged 40–64 years.

*Costs have been inflation-adjusted to 2010 dollars and adjusted for Area Wage Index.

CBSA, Core Based Statistical Area.

Source: AHRQ, Center for Delivery, Organization and Markets from 46 states (AK, AR, AZ, CA, CO, CT, FL, GA, HI, IA, IL, IN, KS, KY, LA, MA, MD, ME, MI, MN, MO, MS, MT, NC, NE, NH, NJ, NM, NV, NY, OH, OK, OR, PA, RI, SC, SD, TN, TX, UT, VA, VT, WA, WI, WV, WY).

**Table 2:** **Average CBSA population and market characteristics, 2005 and 2010**

	All payers	
	2005	2010
***Population characteristics***		
*Unemployment rate*		
Age16+ unemployed (%)	7.2	10.9
*Education*		
Age 25+ with Bachelors or more education (%)	23.5	23.9
*Income*		
Families with income below poverty (%)	10.7	11.8
Household income (mean)	55,755	59,666
Gini index	0.44	0.44
*Population size*		
Total population (n)	558,109	563,555
Population density (population per sq mile) (n)	242.9	244.9
*Race/ethnicity*		
White (%)	76.3	75.1
Black (%)	8.7	9.0
Hispanic (%)	9.8	10.5
Medicaid expenditures per beneficiary	4848.1	5521.3
***Market characteristics***		
Herfindahl-Hirshman Index (0–100)	88.8	88.8
Discharges from a different CBSA (%)	0.20	0.22
Number of acute care hospital beds per 1000 capita (n)	1.6	1.6
Number of long-term care beds per 1000 capita (n)	0.2	0.1
Number of rehabilitation beds per 1000 capita (n)	0.1	0.1
Number of primary care MDs per 100,000 capita (n)	186.6	183.2
Emergency department visits per capita (n)	0.4	0.5
Proportion of acute care beds in high-technology hospitals (%)	0.2	0.3
**Hospital compare quality composite score**		
Heart attack (AMI) (composite score)	90.7	97.8
Heart failure (composite score)	73.2	94.4
Pneumonia (composite score)	79.5	94.9
CBSA, Core Based Statistical Area		

### Empirical strategy

We calculated descriptive statistics and frequency distributions for all of the variables. We then examined the association between unemployment rate and inpatient cost per discharge using a panel regression model. The Hausman specification test was used to determine whether a fixed-effects or random-effects model was more appropriate for the regression analyses [18]. We proceeded with fixed-effects when *p* < .05. The regression models were run for all-payers and then separately by primary expected payer because of the underlying population differences between Medicare and commercial discharges.

We repeated the analyses using an instrumental variable (IV) two stage least squares regression model (2SLS) with fixed- or random-effects because unemployment rate is possibly endogenous due to unobservable confounders such as patient health status that might affect both the unemployment rate and inpatient cost per discharge. Estimates based on the panel model would be biased and inconsistent. The IV analysis provides a consistent estimate of the parameter of interest by generating exogenous variation in the unemployment rate through the use of an instrument that does not lead to any changes in the inpatient cost per discharge [19]. The IV regression also estimates the local average treatment effect as opposed to the average treatment effect.

We used several macro-level variables to exploit exogenous variation and instrument for unemployment rate. In the first stage, we instrumented the unemployment rate on poverty level, mean household income, Gini index, percentage of the population with a Bachelor’s degree or higher, total population per square mile, and an indicator variable as a structural break for the period of market decline for the years after 2007. These macro-level variables were used as instruments because they were highly correlated with the unemployment rate and are assumed to influence the inpatient cost per discharge through the unemployment rate. We tested the strength of the instruments with the F-test before proceeding with the second stage. In the second stage, we used the predicted values of unemployment rate generated from the first stage to fit our regression model. As a post-estimation check, the Sargan statistic was used to test that the overidentification restriction of all instruments was not rejected. The *p*-value was > .05 so we accepted the null hypothesis that our models were not overidentified. This study included the use of de-identified data and did not require Institutional Review Board approval because it does not involve research on human subjects.

## Results

### Description of CBSA characteristics

From the CBSA-level patient characteristics, in 2005, the average age of Medicare discharges was 77.9 years while the average age of commercial discharges was 53.0 years (Table 1). Based on the population and market characteristics, in 2005, the average proportion of Whites was 76.3% while the proportion of discharges from a different CBSA was 0.2%. The average Hospital Compare quality composite scores for heart attack, heart failure, and pneumonia also improved over time (Table 2).

### Changes in unemployment rate and inpatient cost per discharge

**Figure 1:**
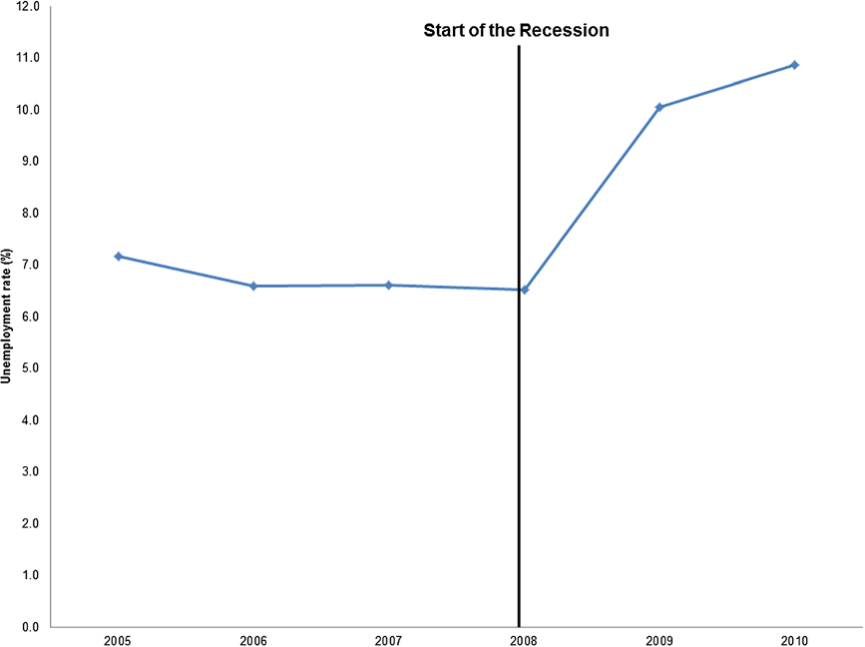
**Average annual CBSA-level unemployment rate, 2005–2010 CBSA, core based statistical area.**

As was expected, the average annual CBSA-level unemployment rate increased substantially over the study period. In 2005, the average unemployment rate was 7.2% and it increased to 10.9% in 2010 (Figure 1).

After adjusting for inflation, the average CBSA-level *total inpatient cost* increased by 10.5% over the study period ($449.5 million to $496.9 million) (data not shown). For Medicare discharges, the average CBSA-level total inpatient cost grew by 5.9% ($224.5 million to $237.7 million) while for commercial discharges it increased by 5.7% ($102.1 million to $107.9 million) (data not shown).

**Figure 2:**
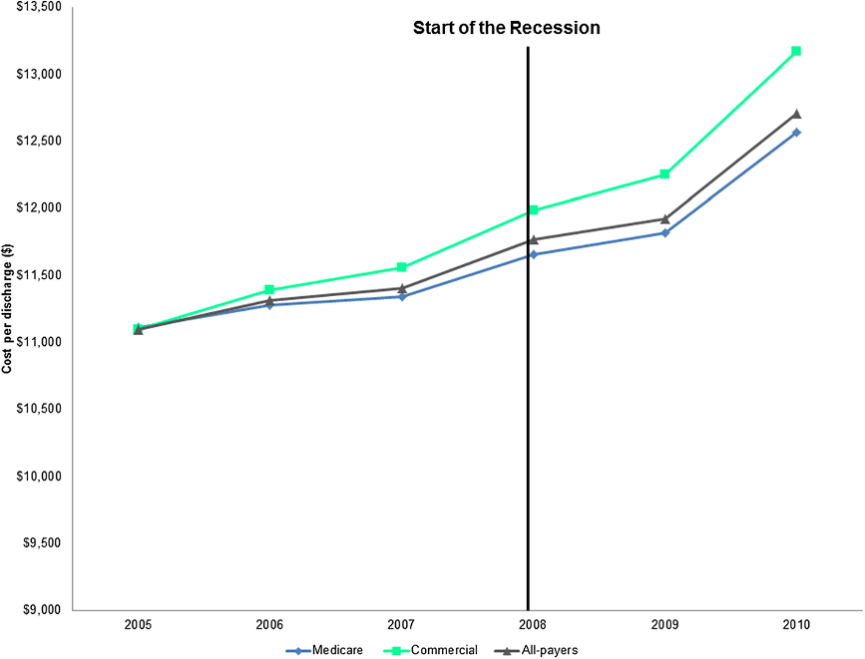
Average annual CBSA-level cost per discharge by payer, 2005-2010* ^**†**^ **.** *Costs have been inflation-adjusted to 2010 dollars and adjusted for Area Wage Index. ^†^Commercial includes non-maternal adults aged 40 to 64 years. CBSA, Core Based Statistical Area.

Although the average CBSA-level *total inpatient cost* grew modestly over time, the average increase in the *cost per discharge* was much larger. Among all-payers, the average inpatient cost per discharge increased by 15% from $11,097 to $12,712, after adjusting for inflation. For Medicare, the average inpatient cost per discharge increased by 13% ($11,110 to $12,570), while for the commercially insured the average inpatient cost per discharge increased by 19% ($11,099 to $13,173), after adjusting for inflation (Figure 2).

### Association between unemployment rate and inpatient cost per discharge

Based on the panel regression model, higher unemployment was positively associated with the inpatient cost per discharge for all-payers and Medicare discharges, but was negatively associated with the inpatient cost per discharge for commercial discharges. None of these associations however, were statistically significant (*p* > .05) (Table 3).

**Table 3:** **Panel regression estimates of CBSA-level unemployment and cost per discharge by primary expected payer with CBSA fixed-effects, adjusted for patient, population, and market characteristics, 2005-2010** ^**†§**^

	All-payer	Medicare	Commercial
	Hausman test *p* < .05	Hausman test *p* < .05	Hausman test *p* < .05
	Fixed-effects	Fixed-effects	Fixed-effects
	n = 2,461	n = 2,461	n = 2,461
	(459 CBSAs and 6 time periods)	(459 CBSAs and 6 time periods)	(459 CBSAs and 6 time periods)
***Unemployment rate***	**Coeff**	**Coeff**	**Coeff**
Age16+ unemployed (%)	20.53	33.40	−3.57

Source: AHRQ, Center for Delivery, Organization and Markets from 46 states (AK, AR, AZ, CA, CO, CT, FL, GA, HI, IA, IL, IN, KS, KY, LA, MA, MD, ME, MI, MN, MO, MS, MT, NC, NE, NH, NJ, NM, NV, NY, OH, OK, OR, PA, RI, SC, SD, TN, TX, UT, VA, VT, WA, WI, WV, WY).

^†^Commercial includes non-maternal adults aged 40 to 64 years.

^§^Adjusted for Area Wage Index.

Coefficients in bold indicate *p* < .05.

From the first stage of the IV regression model, the F-test of the instruments was large and highly significant (F-test = 444.94, *p <* .001) indicating that the instruments were strong. In the second stage of the IV regression, the marginal effect of a one percentage point increase in the unemployment rate was associated with a $47 increase in the inpatient cost per discharge for all-payers (*p* = .032). Similarly, the marginal effect of a one percentage point increase in the unemployment rate was associated with a $37 increase for commercial discharges and a $49 increase for Medicare discharges (*p* = .027 and *p* = .033, respectively) (Table 4).

**Table 4:** **Instrumental variable two stage least squares regression estimates of CBSA-level unemployment and cost per discharge by primary expected payer with CBSA random- or fixed-effects, adjusted for patient, population, and market characteristics 2005-2010** ^**†§**^

	All-payer	Medicare	Commercial
	Hausman test *p* < .05	Hausman test *p >* .05	Hausman test *p* < .05
	Fixed-effects	Random-effects	Fixed-effects
	n = 2,461	n = 2,461	n = 2,461
	(459 CBSAs and 6 time periods)	(459 CBSAs and 6 time periods)	(459 CBSAs and 6 time periods)
***Unemployment rate***	**Coeff**	**Coeff**	**Coeff**
Age16+ unemployed (%)	**47.02**	**48.61**	**37.44**

Source: AHRQ, Center for Delivery, Organization and Markets from 46 states (AK, AR, AZ, CA, CO, CT, FL, GA, HI, IA, IL, IN, KS, KY, LA, MA, MD, ME, MI, MN, MO, MS, MT, NC, NE, NH, NJ, NM, NV, NY, OH, OK, OR, PA, RI, SC, SD, TN, TX, UT, VA, VT, WA, WI, WV, WY).

^†^Commercial includes non-maternal adults aged 40 to 64 years.

^§^Adjusted for Area Wage Index.

Coefficients in bold indicate *p* < .05.

## Discussion

In our analyses of HCUP data from 2005–2010, we find evidence that the inpatient cost per discharge is countercyclical – unemployment is associated with an *increase in the cost per discharge* for all-payers and payer-specific discharges (Medicare and commercial). The magnitude of this effect however, was stronger for the Medicare population. Additionally, although previous reports have linked unemployment with lower hospital utilization, we find that unemployment is associated with a higher inpatient cost per discharge.

Our study builds upon previous work by examining the effect of one recent economic downturn on the changes in hospital resource use for different segments of the population. Prior studies have suggested that individuals who are continuously insured during economic recessions might reduce their demand for routine medical care and defer treatment [6, 20, 21]. Continuously insured individuals might reduce discretionary spending on health care services and take fewer preventive measures because of the fear of potential job loss, increased economic uncertainty, and greater financial stress. Over prolonged periods, this may result in deteriorating health status and greater downstream utilization of more costly services [22]. This finding is consistent with a May 2009 survey of family physicians who reported seeing patients with more health problems caused by forgone preventive care [22]. Similarly, a survey among employed individuals with employer-sponsored insurance found that individuals chose not to seek medical treatment to save money on co-payment or co-insurance and had skipped taking medications at prescribed doses [23]. Thus, the increased cost per discharge among the commercially insured might stem from more resource intensive services used by patients who postponed their medical care.

Our study also provides evidence of a significant relationship between the unemployment rate and increased cost per discharge for the Medicare population. A possible explanation for this finding is that there may be changes in the utilization pattern and resource use among Medicare beneficiaries during periods of high unemployment. From the supply-side, providers who are made financially worse off during a recession because of lower demand by the commercially insured might have stronger financial incentives and greater capacity to treat Medicare patients [24]. Providers might be more willing to accommodate Medicare patients during economic slowdowns, which would lead to higher spending. Consistent with this hypothesis, evidence from the Community Tracking Study Physician Survey supports the notion that physicians are more willing to accept new Medicare patients during periods of high state unemployment [5]. Thus, the underlying mechanisms by which unemployment might drive the growth in inpatient costs may differ between payer groups.

There are some limitations to our study. Because of the ecological nature of the study design, we do not know if patients with costly discharges are more likely to be unemployed. Also, because our study only focused on the cost per discharge for all discharge types there may be different effects of the unemployment rate for specific clinical conditions, such as ambulatory care sensitive conditions and elective procedures. There may also be other unmeasured characteristics that might have influenced our findings. Other possible factors that may have contributed to the increase in the inpatient cost per discharge over the study period include the adoption of new and expensive medical technologies as well as an older, sicker patient population. To address this, we controlled for possible confounding by including the proportion of acute care beds in high technology hospitals and adjusted our models for patient age and comorbidities. Lastly, our study only includes data from one recent economic downturn.

The study limitations are countered by some design strengths. We used an instrumental variables approach to examine the effect of changes in unemployment rate on the inpatient cost per discharge. The IV approach permits us to have stronger causal inferences and unbiased estimates of the parameter of interest. We also controlled for a number of patient, population, and market characteristics that may affect resource use. Another strength is that we used HCUP SID from 46 states. Although there are no data sources that include all population groups and all health care settings, our findings confirm that readily available electronic data such as HCUP can be useful in studying changes in inpatient resource use across multiple payers. We also examined variations at the CBSA-level because there might be greater variation in unemployment and costs at the small-area.

## Conclusions

In conclusion, we find evidence that the inpatient cost per discharge is countercyclical for Medicare and commercial discharges. The underlying mechanism by which unemployment affects hospital resource use however, might differ between payer groups. Further exploration is needed to better understand the contributors to the increase in inpatient cost per discharge during periods of economic contractions.

## Authors’ information

JLM was a research scientist at the Kaiser Permanente, Mid-Atlantic Permanente Research Institute. RMH is a director at Truven Health Analytics. WDM is a senior vice president at Truven Health Analytics. ZK is a senior health economist at the Agency for Healthcare Research and Quality. BSF is a retired senior health economist at the Agency for Healthcare Research and Quality. HSW is a senior health economist at the Agency for Healthcare Research and Quality.

## Abbreviations

2SLS: Two stage least squares
ACS: American Community Survey
AHA: American Hospital Association
AHRF: Area Health Resource file
CBSA: Core based statistical area
CMS: Centers for Medicare and Medicaid Services
ED: Emergency department
FFS: Fee-for-service
HCUP: Healthcare Cost and Utilization Project
HHI: Herfindahl-Hirschman Index
HMO: Health maintenance organization
HMS: Hospital market structure
HRR: Hospital referral region
IV: Instrumental variables
POS: Point-of-service
PPO: Preferred provider organization
SID: State Inpatient Databases.
